# Optimal Surgical Margin After Breast-Conserving Surgery in Pure Ductal Carcinoma in Situ: Is a 1 mm Margin Sufficient? A Retrospective Single-Center Cohort Study

**DOI:** 10.3390/medicina62061061

**Published:** 2026-05-31

**Authors:** Ufuk Karabacak, Murat Derebey, Ismail Alper Tarim, Saim Savas Yuruker, Bahadir Bulent Gungor, Ayfer Kamali Polat

**Affiliations:** Department of General Surgery, Faculty of Medicine, Ondokuz Mayıs University, Samsun 55200, Türkiye

**Keywords:** ductal carcinoma in situ, breast-conserving surgery, surgical margin width, ipsilateral breast tumor recurrence, re-excision

## Abstract

*Background/Objectives:* The optimal surgical margin width after breast-conserving surgery (BCS) for pure ductal carcinoma in situ (DCIS) remains controversial; although current guidelines consider a surgical margin of ≥2 mm sufficient, the clinical safety of narrower margins is unclear. This study aimed to evaluate the association between surgical margin width and ipsilateral breast tumor recurrence (IBTR), with a focus on the 1 mm threshold. *Materials and Methods:* In this retrospective single-center cohort study, 107 patients with pure DCIS treated with BCS followed by adjuvant radiotherapy (RT) between 1 January 2009 and 1 January 2025 were analyzed. Final surgical margins were categorized as <1 mm, 1–2 mm, and ≥2 mm. The primary endpoint was IBTR. Kaplan–Meier analysis was performed. *Results:* The median age of the study population was 52 years (IQR: 46–61). High-grade DCIS was present in 48 patients (44.9%), comedo necrosis in 68 (63.6%), and estrogen receptor positivity in 87 (81.3%). Overall, 10 patients (9.3%) underwent re-excision for margin widening. Final surgical margin widths were <1 mm in 36 patients (34%), 1–2 mm in 18 (17%), and ≥2 mm in 53 (49%). IBTR occurred in seven patients (6.5%) during a median follow-up of 48 months (range, 12–217 months), with a median time to recurrence of 33 months. Kaplan–Meier analysis showed no significant difference in recurrence-free survival according to a 2 mm margin threshold, whereas margins < 1 mm were associated with significantly worse outcomes (*p* = 0.002). *Conclusions:* Margins < 1 mm were associated with increased IBTR risk, whereas margins < 2 mm did not appear to confer uniform risk. These findings suggest that margin widths between 1 and 2 mm may represent a heterogeneous group, and clinical decision-making in this range should be individualized. However, further studies are needed to validate these outcomes.

## 1. Introduction

Ductal carcinoma in situ (DCIS) is a preinvasive breast cancer classified as stage 0 [1,2]. Although DCIS is defined as a localized disease with no metastatic potential, it can transform into invasive carcinoma in 25–60% of cases if left untreated [1]. Furthermore, invasive tumors can be detected in surgical excision materials of patients diagnosed with DCIS via core needle biopsy [3]. These factors necessitate meticulous planning and an individualized treatment approach.

The primary treatment for DCIS is surgical excision of the tumor. Other components of multidisciplinary treatment include radiotherapy and endocrine therapy, which are administered according to the surgical procedure performed, hormone receptor status, and individualized recurrence risk [4,5,6,7]. IBTR rates of up to 20% have been reported despite adjuvant RT in patients undergoing breast-conserving surgery (BCS) [8,9]. Approximately half of IBTRs present as invasive tumors, and the rate of regional or distant metastasis reaches 6% in patients with invasive tumor recurrence after BCS [10,11]. Although DCIS is regarded as a localized disease, IBTR and progression to invasive carcinoma remain clinically significant challenges.

Factors such as surgical margin width, young age, family history, clinical presentation, and tumor grade have been found to be associated with IBTR [12]. In contrast to largely non-modifiable patient- and tumor-related factors, surgical margin width is uniquely significant because it is directly modifiable at the time of surgery. Initially, quite wide surgical margins of 1 cm were considered reasonable, but over time, with the addition of RT to BCS, a surgical margin width of 2 mm has been accepted as sufficient [12,13]. Current guidelines recommend a minimum surgical margin of 2 mm [4,5,6,7]. Therefore, many surgeons tend to perform re-excision at closer surgical margins. However, the presence of studies suggesting that surgical margins closer than 2 mm are also oncologically acceptable and do not increase the risk of recurrence renders the issue controversial [9,12,14].

Even a change of only 1 mm in the optimal surgical margin can significantly affect the number of possible re-excisions [12,15,16,17]. While the adequate surgical margin width remains controversial, consideration of the psychosocial, financial, and cosmetic effects of additional surgical interventions raises concerns regarding whether routine re-excision may constitute overtreatment. Furthermore, in selected patients, aggressive attempts to achieve margins wider than 2 mm may lead to unnecessary conversion to mastectomy, thereby compromising the cosmetic, psychological, and quality-of-life benefits of breast-conserving surgery.

In this study, we aimed to investigate the optimal surgical margin width in patients with pure DCIS who underwent BCS and to directly compare 1 mm and 2 mm surgical margin thresholds with respect to IBTR risk.

## 2. Materials and Methods

### 2.1. Study Design and Patient Selection

After obtaining approval from the Ondokuz Mayıs University Clinical Research Ethics Committee (approval number: 2025/442), patients who underwent BCS with a diagnosis of DCIS at the General Surgery Clinic between 1 January 2009 and 1 January 2025 were retrospectively reviewed. Patients were eligible for inclusion if they had histopathologically confirmed pure DCIS, were treated with breast-conserving surgery followed by adjuvant radiotherapy, and had available clinicopathological and follow-up data for at least 12 months. Eligibility assessment was performed according to predefined inclusion and exclusion criteria by one investigator and independently verified by a second investigator to minimize selection bias.

Patients were excluded if they had:

- a prior history of breast cancer or other malignancy,
- invasive or microinvasive components on final pathological examination,
- conversion to mastectomy during reoperation,
- follow-up shorter than 12 months, or
- incomplete clinical data
- male sex.

### 2.2. Data Collection and Variables

Demographic, clinicopathological, radiological, and treatment-related data were systematically extracted from medical records. The following variables were analyzed: age, surgical margin width, recurrence-free follow-up period, IBTR, pathological tumor diameter, presence of microcalcifications on mammography, use of preoperative magnetic resonance imaging (MRI), lesion focality (unifocal/multiple), pathological diagnostic method (core needle biopsy vs. excisional biopsy), preoperative wire-guided lesion localization, re-excision status, sentinel lymph node biopsy (SLNB), presence of comedo necrosis, tumor grade, number of pathological foci, and estrogen and progesterone receptor status. IBTR was defined as any histopathologically confirmed ipsilateral in situ or invasive breast tumor recurrence after BCS. Radiologically suspected lesions were not considered IBTR unless pathological confirmation was obtained. The final surgical margin was defined as the margin of the initial surgery in patients without re-excision and as the margin of the last surgery in those undergoing re-excision. Estrogen and progesterone receptor positivity was defined as ≥1% nuclear staining.

### 2.3. Surgical, Pathologic, and Adjuvant Treatment Protocols

Radiologically suspicious lesions were evaluated preoperatively for core needle biopsy. Excisional biopsy was performed in patients not suitable for core needle biopsy or in those in whom radiologic-pathologic discordance was identified after biopsy. Breast-conserving surgery was performed with the aim of complete removal of the radiologically or clinically identified lesion with negative surgical margins while preserving acceptable breast cosmesis. Depending on lesion location, breast size, anticipated resection volume, and expected cosmetic outcome, surgery was performed either as standard wide local excision/lumpectomy or, in selected patients, using oncoplastic breast-conserving techniques. For palpable lesions, excision was guided by clinical findings, whereas non-palpable lesions were excised after preoperative wire-guided localization performed by the radiology department, with concurrent marking of the skin projection. The lesion was removed en bloc with surrounding breast tissue. In lesions located close to the skin, the overlying skin was included in the excision when necessary to obtain an adequate anterior surgical margin. When anatomically appropriate, the deep surgical margin was extended to the pectoral fascia. Surgical specimens were oriented and sent fresh to the pathology laboratory before formalin fixation to allow proper margin assessment. In patients with microcalcifications, specimen radiography was used to confirm removal of the targeted radiological abnormality. Additional margin excision was performed intraoperatively when the surgeon considered the macroscopic margin inadequate or when specimen radiography suggested insufficient excision. In standard lumpectomy cases, the cavity was closed primarily after limited glandular approximation when necessary. Routine cavity shaving was not performed. At our institution, adjuvant RT is recommended for all patients undergoing BCS due to DCIS, and hormone therapy is recommended for all hormone receptor-positive patients.

### 2.4. Statistical Analysis

All statistical analyses were performed using IBM SPSS Statistics for Windows, version 27.0 (IBM Corp., Armonk, NY, USA). The distribution of continuous variables was evaluated using the Shapiro–Wilk test. Since continuous variables did not exhibit a normal distribution, continuous variables were summarized using the median and interquartile range (IQR), and the Mann–Whitney U test was used for intergroup comparisons. The Fisher exact test was applied for comparisons of categorical variables. Time-to-event analyses were performed using the Kaplan–Meier method, and survival curves were compared using the log-rank test. Time to recurrence was defined as the interval from surgery to IBTR, and patients without recurrence were censored at the date of last follow-up. The median follow-up time was estimated using the reverse Kaplan–Meier method. Among patients who developed recurrence, time to recurrence was summarized using the median and IQR. Considering the limited number of recurrence events, multivariable modeling, including logistic regression and Cox proportional hazards analysis, was not undertaken to avoid model overfitting and instability of parameter estimates. All tests were two-sided, and a *p*-value < 0.05 was considered statistically significant.

## 3. Results

**Figure 1:**
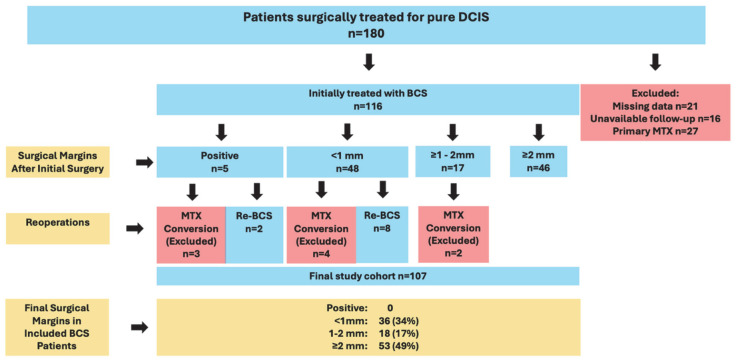
Flow diagram of patient selection and surgical margin distribution.

A total of 180 patients with pure DCIS treated surgically at our institution were reviewed. Twenty-seven patients who underwent primary mastectomy, 37 patients with unavailable follow-up or insufficient clinical/pathological data, and 9 patients who required conversion to mastectomy after BCS due to surgical margin status were excluded (Figure 1).

A total of 180 patients surgically treated for pure DCIS were evaluated. Patients were excluded because of missing data (*n* = 21), unavailable follow-up (*n* = 16), or primary mastectomy (*n* = 27). Among 116 patients initially treated with BCS, 9 were excluded because of conversion to mastectomy after reoperation for margin status. The final study cohort consisted of 107 patients with final BCS. The lower panel summarizes the final surgical margin distribution in the included patients. BCS: breast-conserving surgery; DCIS: ductal carcinoma in situ; MTX: mastectomy.

A total of 107 patients were included. The median age of the study population was 52 years (IQR: 46–61). The median follow-up, estimated using the reverse Kaplan–Meier method, was 48 months (95% CI: 30–66; range, 12–217 months), and 43% of patients had at least 5 years of follow-up. Among patients who developed recurrence, the median time to recurrence was 33 months (IQR: 23–62). Overall, 10 patients (9%) underwent re-excision for margin widening. All analyses of surgical margin width were based on the final surgical margin status after completion of all surgical procedures; therefore, patients who underwent re-excision were classified according to the margin width achieved after re-excision. No IBTR was observed among patients who underwent re-excision, and re-excision status was not significantly associated with IBTR (*p* = 0.493). Final surgical margin widths were <1 mm in 36 patients (34%), 1–2 mm in 18 patients (17%), and ≥2 mm in 53 patients (49%) (Figure 1). Adjuvant RT was administered to all included patients. Overall, IBTR was detected in 7 patients (6.5%). Among the seven patients with IBTR, three had invasive ductal carcinoma recurrence, whereas four had pure DCIS recurrence. Five recurrences occurred in the original tumor bed, whereas two occurred elsewhere in the ipsilateral breast, away from the original tumor bed. None of the patients who developed IBTR had undergone re-excision after the initial surgery. Six of the seven IBTR events, including all three invasive recurrences, occurred in patients with final surgical margins < 1 mm. The remaining patient had a final surgical margin ≥ 2 mm and presented with Paget disease recurrence without an isolated radiological mass. Among the 14 patients with pathologically multifocal DCIS, final surgical margins were <1 mm in seven patients and ≥2 mm in seven patients. The only recurrence observed among patients with multifocal DCIS occurred in a patient with a final surgical margin < 1 mm. No distant metastases were observed.

The comparison of clinicopathological, radiological, and treatment-related characteristics according to IBTR status is presented in Table 1. When classified according to final surgical margin width as <2 mm and ≥2 mm, no significant association was observed between margin width and IBTR (*p* = 0.114). However, when reclassified as <1 mm and ≥1 mm, margins <1 mm were significantly associated with IBTR (*p* = 0.007). High tumor grade (*p* = 0.002), absence of preoperative wire-guided lesion localization (*p* = 0.028), and performance of sentinel lymph node biopsy (SLNB) (*p* = 0.017) were also significantly associated with IBTR. No significant associations were observed between IBTR and age, pathological tumor diameter, presence of microcalcifications on mammography, pathological or radiological focality, use of preoperative MRI, re-excision status, diagnostic biopsy method, presence of comedo necrosis, or estrogen and progesterone receptor status.

**Figure 2:**
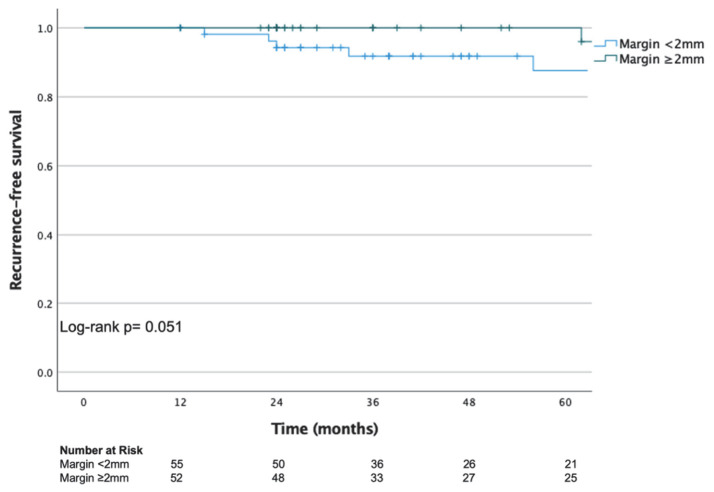
Kaplan–Meier analysis of recurrence-free survival according to surgical margin < 2 mm versus ≥2 mm.

**Figure 3:**
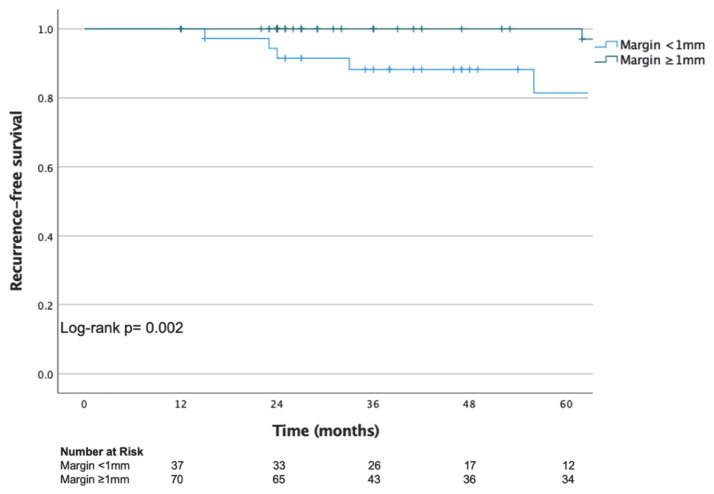
Kaplan–Meier analysis of recurrence-free survival according to surgical margin < 1 mm versus ≥1 mm.

In Kaplan–Meier analysis, no significant difference in recurrence-free survival was observed between patients with margins < 2 mm and ≥2 mm (log-rank test, *p* = 0.051) (Figure 2). In contrast, when stratified as <1 mm versus ≥1 mm, recurrence-free survival was significantly worse in patients with margins < 1 mm (log-rank test, *p* = 0.002) (Figure 3).

## 4. Discussion

Although there is ongoing debate regarding the optimal management of DCIS, surgery remains the gold standard of treatment. Surgical options include simple mastectomy, BCS, and nipple–areola- or skin-sparing procedures. With the maturation of multidisciplinary treatment strategies, BCS has become the preferred approach for many patients. However, the definition of an adequate surgical margin to achieve optimal oncologic outcomes has evolved over time. In earlier periods, wider surgical margins were targeted, whereas with the incorporation of adjuvant RT, narrower margins were considered sufficient [12]. Current guidelines, including those from the Association of Breast Surgery (ABS), the American Society of Breast Surgeons (ASBrS), the National Comprehensive Cancer Network (NCCN), and the European Society for Medical Oncology (ESMO), consider a 2 mm margin adequate in patients undergoing BCS and recommend adjuvant radiotherapy and endocrine therapy according to hormone receptor status [4,5,6,7]. Consequently, many surgeons consider re-excision or conversion to mastectomy when margins are <2 mm at the initial surgery.

However, the 2016 Society of Surgical Oncology–American Society for Radiation Oncology–American Society of Clinical Oncology (SSO–ASTRO–ASCO) consensus guideline, which has been cited in major contemporary guidelines including ABS, ASBrS, NCCN, and ESMO, concluded that a 2 mm margin is sufficient to minimize IBTR and recommended that re-excision for margins < 2 mm be selectively applied [18]. Despite this, several studies comparing re-excision rates before and after implementation of the SSO–ASTRO–ASCO consensus guideline have reported no significant reduction, or even an increase, in reoperation rates [15,19]. Thus, adopting a selective rather than routine re-excision approach for patients with margins <2 mm has not consistently reduced reoperation rates. This discrepancy suggests that, although selective re-excision is recommended by the guideline, the lack of clearly defined criteria may limit its consistent implementation in routine clinical practice.

Furthermore, the rate of close but negative (<2 mm) margins after BCS ranges from 17% to 41%, and reoperation rates of up to 60% have been reported in this subgroup [12,15,16,17]. In an effort to achieve the targeted margin width, some patients are converted to mastectomy, whereas others may require a third surgical intervention [15,16]. The potential cosmetic, psychosocial, and financial consequences of repeated surgeries should not be underestimated [20,21]. Achieving a balance between optimal oncologic control and avoidance of overtreatment remains a key clinical priority.

In a study evaluating the impact of surgical margin width, Van Zee et al. categorized patients undergoing BCS according to margin status (positive, <2 mm, >2–10 mm, and >10 mm) and found that wider margins were associated with a significantly lower IBTR rate in patients who did not receive RT, whereas no significant association between margin width and IBTR was observed among those receiving RT [12]. Similarly, Tadros et al., comparing patients with margins < 2 mm and ≥2 mm, reported no difference in IBTR among those receiving RT and concluded that routine re-excision in patients with margins < 2 mm may not be justified [9]. Most studies derive their conclusions by classifying patients according to the 2 mm threshold. Data specifically addressing clear but very close margins, such as 1 mm, remain limited. Accordingly, margins between 1 and 2 mm, which comprise a significant subset of cases considered for re-excision, remain a clinical gray zone. This underscores the relevance of clarifying whether a 1 mm surgical margin can be regarded as sufficient.

Notably, a study incorporating a 1 mm threshold by Ekatah et al. categorized patients into three groups (<1 mm, 1–2 mm, and >2 mm) and found no difference in IBTR between the 1–2 mm and >2 mm groups, concluding that a 1 mm margin may be adequate in DCIS patients undergoing BCS [14]. In our study, when patients were classified as <2 mm versus ≥2 mm, no significant difference in IBTR was observed (Table 1, Figure 2). However, when the same cohort was reclassified according to a <1 mm versus ≥1 mm threshold, margins < 1 mm were associated with a significantly increased risk of IBTR (Table 1, Figure 3). These findings suggest that a 1 mm margin may provide better discrimination than the conventional 2 mm standard. Another clinically important consideration is the biological and prognostic significance of recurrent disease. In our cohort, three of the seven IBTR events (42.9%) presented as invasive ductal carcinoma, and all invasive recurrences occurred in patients with final surgical margins < 1 mm. Because invasive recurrence carries metastatic potential, these observations may further support the clinical relevance of distinguishing very close margins from wider negative margins, rather than considering all margins < 2 mm as a single homogeneous risk category.

Although inconsistent findings have been reported in the literature, tumor grade was significantly associated with IBTR in our cohort, and all recurrences occurred in high-grade DCIS cases [2,9,12]. High-grade disease, palpable presentation, larger tumor extent, comedo necrosis, and, in some studies, HER2 positivity have been described as features associated with more aggressive DCIS biology and a higher likelihood of occult invasion or upstaging at final pathology [22,23]. Therefore, SLNB may be considered in selected patients with biopsy-proven DCIS who have high-risk clinical, radiological, or pathological features, particularly when the probability of invasive disease in the final excision specimen is considered substantial [23]. When SLNB is indicated, sentinel nodes can be identified using conventional blue dye, radioisotope-based mapping, or indocyanine green fluorescence, whereas frozen-section examination and emerging optical imaging or spectroscopic techniques may be used for intraoperative assessment of metastatic involvement in the excised lymph nodes [23,24,25]. In our cohort, SLNB was not performed routinely; rather, it was reserved for patients with preoperatively recognized aggressive features and was performed using blue dye with intraoperative frozen-section assessment. Preoperative wire-guided localization was used only for non-palpable lesions. Therefore, the observed associations between IBTR and both SLNB and the absence of preoperative wire-guided localization should not be interpreted as causal effects of these procedures. Rather, they most likely reflect confounding by indication: SLNB was preferentially performed in patients with higher-risk disease, whereas the absence of wire-guided localization likely represented clinically evident lesions and, consequently, a higher baseline risk profile.

The retrospective, single-center design and the relatively limited sample size represent the main methodological limitations of our study. Nevertheless, the inclusion of a homogeneous cohort composed exclusively of patients who underwent BCS followed by adjuvant RT, together with the focused evaluation of a 1 mm surgical margin threshold that has often been overlooked in previous studies, provides a more refined perspective on the ongoing debate regarding optimal margin width. In this context, our findings should be considered hypothesis-generating and may support more individualized decision-making in clinical practice.

## 5. Conclusions

Findings from this study suggest that surgical margins < 2 mm do not constitute a uniform risk group in patients with pure DCIS undergoing BCS, whereas margins < 1 mm were associated with increased IBTR risk. These results indicate that margin widths between 1 and 2 mm may represent a heterogeneous group, and clinical decision-making in this range may benefit from individualized assessment. Further studies are needed to validate these findings.

## Figures and Tables

**Table 1:** Factors Associated with Ipsilateral Breast Tumor Recurrence.

	No IBTR *n* (%)	IBTR *n* (%)	*p* Value
Age (median [IQR], years)	54 (46–62)	47(39–50)	0.071
Microcalcification on Mammography			
Presence	75(93)	6(7)	
Absence	25(96)	1(4)	0.457
Preoperative MRI			
Yes	32(100)	0	
No	68(91)	7(9)	0.100
Radiologic Focality			
Single	94(94)	6(6)	
Multiple	6(86)	1(14)	0.386
Diagnostic Biopsy Method			
Core Needle	59(92)	5(8)	
Excisional	41(95)	2(5)	0.699
Preoperative Lesion Marking			
Yes	72(97)	2(3)	
No	28(85)	5(15)	**0.028**
Tumor Diameter (median [IQR], mm)	15(8–35)	19 (13–60)	0.231
Tumor Grade			
1–2	59(100)	0	
3	41(85)	7(15)	**0.003**
Comedo Necrosis			
Presence	62(91)	6(9)	
Absence	38(97)	1(3)	0.418
Pathologic Focality			
Single	87(93)	6(7)	
Multiple	13(93)	1(7)	0.637
Estrogen Receptor Status			
Positive	82(94)	5(6)	
Negative	18(90)	2(10)	0.613
Progesterone Receptor Status			
Positive	71(96)	3(4)	
Negative	29(88)	4(12)	0.199
Margin Re-excision			
Yes	10(100)	0	
No	90 (93)	7(7)	0.493
SLNB			
Yes	53(88)	7(12)	
No	47(100)	0	**0.017**
Final Surgical Margin Width (<1 mm vs. ≥1 mm)			
<1 mm	31 (84)	6 (16)	
≥1 mm	69 (99)	1 (1)	**0.007**
Final Surgical Margin Width (<2 mm vs. ≥2 mm)			
<2 mm	49(89)	6(11)	
≥2 mm	51(98)	1(2)	0.114

IBTR, ipsilateral breast tumor recurrence; IQR, interquartile range; SLNB, sentinel lymph node biopsy; MRI, magnetic resonance imaging. Bold values indicate statistical significance.

## Data Availability

The datasets used and/or analyzed during the current study are available from the corresponding author on reasonable request.

## Abbreviations

ABS	Association of Breast Surgery
ASBrS	American Society of Breast Surgeons
BCS	Breast-conserving surgery
CI	Confidence interval
DCIS	Ductal carcinoma in situ
ESMO	European Society for Medical Oncology
IBTR	Ipsilateral breast tumor recurrence
IQR	Interquartile range
MRI	Magnetic resonance imaging
NCCN	National Comprehensive Cancer Network
OR	Odds ratio
RT	Radiotherapy
SLNB	Sentinel lymph node biopsy
SSO–ASTRO–ASCO	Society of Surgical Oncology–American Society for Radiation Oncology–American Society of Clinical Oncology
